# Knowledge, attitudes and practices towards dog-bite related rabies in para-medical staff at rural primary health centres in Baramati, western India

**DOI:** 10.1371/journal.pone.0207025

**Published:** 2018-11-16

**Authors:** Harish Kumar Tiwari, Abi Tamim Vanak, Mark O’Dea, Ian Duncan Robertson

**Affiliations:** 1 College of Veterinary Medicine, School of Veterinary and Life Sciences, Murdoch University, Western Australia, Australia; 2 Ashoka Trust for Research on Ecology and Environment (ATREE), Bangalore, India; 3 Wellcome Trust/DBT India-Alliance, Hyderabad, India; 4 School of Life Sciences, University of KwaZulu-Natal, Durban, South Africa; 5 China-Australia Joint Research and Training Center for Veterinary Epidemiology, Huazhong Agricultural University, Wuhan, Hubei, China; Sciensano, BELGIUM

## Abstract

The lack of awareness regarding rabies amongst rural primary care health staff and their adverse practices towards the management of dog-bite wounds is a major contributor to the high incidence of rabies infection and subsequent human mortality in India. A Knowledge, Attitudes and Practices survey was carried out involving 54 nursing and non-nursing staff working in 18 rural Primary Health centres and sub-centres around Baramati town of Pune district in Western India. Multivariable logistic regression models were constructed to assess factors that influenced knowledge of rabies and practices towards management of dog-bite related wounds. The more experienced and better-educated workers were found to have a good awareness of rabies (OR 3.4, 95%CI 1.0–12.1) and good practices towards dog-bite wound management (OR 5.6, 95%CI 1.2–27.0). Surprisingly, non-nursing staff were significantly more knowledgeable about rabies (OR 3.5, 95%CI 1.0–12.3), but their practices towards dog-bite wound management were inadequate (OR 0.18, 95%CI 0.04–0.8) compared to the nursing staff. It is recommended that a mandatory training module for primary care health staff be developed and implemented to improve their knowledge regarding rabies and management of dog-bite wounds to reduce the incidence of human rabies in rural India.

## 1. Background

Rabies is a viral zoonosis transmitted through the bite of a rabid animal and affects all warm blooded animals [1]. Although it is present in most countries of the world [2], the incidence in developing nations is higher, with India contributing more than 36% of global deaths each year [3, 4], of which the majority are as a result of bites from free-roaming dogs. However the impact of rabies in India is likely to be even larger due to an inadequate reporting system [5]. The disease primarily affects disadvantaged groups, in both rural and urban areas, due to a lack of awareness of the disease, insufficient financial resources to seek medical help, poor health care infrastructure, unavailability of prophylactic and therapeutic measures and an overemphasis on the use of traditional practices for treatment and wound healing [6, 7]. The rural population is more likely to suffer higher mortalities due to a lack of infrastructure and staff to provide timely first aid in the form of wound cleaning and administration of post-exposure prophylaxis (PEP) [8].

The role of primary healthcare staff, who are the first point of contact for dog-bite victims seeking medical intervention, is crucial for the prevention of rabies [9]. Although the number of Primary Health Centres (PHC) in rural areas of India is increasing, the presence of sufficient adequately qualified personnel to staff these centres remains a challenge for the Indian government. Although the focus of these health centres is control of preventable diseases of children, such as diphtheria, pertussis, tetanus, measles and poliomyelitis [10], they are also responsible for administering anti-rabies PEP and providing first aid measures for dog-bite victims. Consequently, assessment of the knowledge, attitudes and practices (KAP) of staff towards rabies and animal bites is a key factor in the effective control of rabies [11].

There is a reported lack of adequate knowledge about the preventive measures adopted, including PEP, of primary health care professionals, especially in rural India [9]. Furthermore, there is evidence that some physicians know little about the correct prophylactic measures to adopt to prevent rabies [12], and this lack of adequately trained medical and paramedical staff contributes towards the failure of the rabies control strategy adopted in India [13]. Knowledge, skills and motivation of health care providers are essential for effective prevention and control of diseases; however, India has been unable to meet set targets for endemic diseases [14]. In the case of rabies, it is important to understand the level of knowledge and preparedness of health workers, particularly those from rural areas, to deal with patients who suffer dog-bites.

In rural areas there is often a failure to provide PEP to dog-bite victims in a timely manner due to unavailability at PHC or privately owned pharmacies in these areas. Consequently PEP or Rabies Immunoglobulins (RIG) must be acquired from adjacent urban centres, an option which is often not undertaken or is delayed by patients due to distance, time and/or cost, increasing the likelihood of progression to clinical rabies [15]. These cases could potentially be prevented if the PEP/RIG was either readily available at rural PHC or if health workers at PHC where there was no PEP/RIG available could convince dog-bite victims of the importance of obtaining these products.

We conducted a KAP survey in PHC and sub-centres in the rural areas around the town of Baramati to assess: 1) the KAP of the paramedical staff towards rabies; 2) the availability of PEP/RIG in rural PHC; and 3) the awareness of the rural paramedical staff on the use of PEP/RIG.

## 2. Materials and methods

### 2.1 Study area, sampling procedure and sample size

All 18 operational PHC and sub-centres located within a 20 kilometre radius of Baramati town of Pune district in western India were included in this study. These cater to the primary health needs of approximately 360,000 rural residents (Economic Survey of Maharashtra, 2017–18, http://admin.indiaenvironmentportal.org.in). The centres were visited during 15^th^ to 19^th^ December 2016 and the paramedical staff present at the time of the visit included in the survey. A total of 54 staff members were interviewed from the 18 centres. The respondents comprised of 31 nursing staff (GNM/ANM) and 23 non-nursing staff (Laboratory technicians; Pharmacists; Multi-purpose workers (MPW); Ladies’ Health visitors (LHV); Health workers (HW); On Job Trained workers (OJT)).

### 2.2 Questionnaire design

The KAP survey was designed to determine the awareness of the paramedical staff regarding rabies and to assess practices adopted to prevent and control the disease. The questionnaire consisted of closed questions in two sections: Section one explored demographic details regarding information about the individual pertaining to their medical qualifications, years of experience and if the individual had undergone any training to manage animal bite injuries. The second section was comprised of questions to assess the knowledge, attitudes and practices of the individuals with respect to rabies. The permission of the Taluka health officer, Panchayat Samiti Baramati was obtained to conduct this study. Ethics approval was obtained from the Murdoch University Human Ethics Committee (permission number: 20/2016).

Prior to administering the questionnaire, the study was explained to the participants in the language they understand (Marathi), the confidentiality of their answers confirmed and their signed consent to participate was obtained. No personal details of the participants, including their name, were recorded. The questionnaire was read out to the participant in their local language (Marathi) and the answers were recorded in English.

### 2.3 Data management and analysis

The answers to the questions were tabulated in a spreadsheet (Microsoft Excel, Microsoft Corp., Redmond, WA, USA). Before submitting for statistical analysis, data were made compatible for analyses in the R programming environment [16], which included converting all “Not sure” responses as incorrect and removal of “NA” (not applicable) responses.

A participant’s “knowledge” about rabies and “attitudes and practices” about rabies were the dependent variables in the analyses, while the “years of experience”, “educational level” and the “professional appointment” held at the PHC were the explanatory variables for these analyses. Univariable analyses were initially performed using the Chi-squared test of independence or the Fisher’s exact test. Variables with a p-value ≤ 0.25 were then offered to a multivariable logistic regression model and a final model generated using a backward stepwise process. Variables with a p < 0.05 were retained in the final model. The final model was evaluated with the Hosmer-Lemeshow goodness-of-fit test [17]. Odds ratios were calculated using the “odds ratio” package in R [18].

The answers to six questions were used to assess a participant’s knowledge about rabies and the answers to 10 used to assess their practices. For these questions a correct answer was scored as 1 and an incorrect answer as 0. The results for these questions were summed and the median score for all participants calculated. People with a knowledge score higher than 4 (median score) were categorised as having good knowledge and those ≤ 4 as having poor knowledge. Similarly people with a practices score > 6 (median) were categorised as having positive practices. The division of respondents on the basis of median score on knowledge about rabies and on the practices regarding dog-bite wound management was based on recommendations for assessing Likert type scales [19].

## 3. Results

A total of 54 staff employees were interviewed from 14 PHCs and 4 sub-centres located with 20 km of Baramati (Table 1).

**Table 1:** List of primary health centres and sub-centres around Baramati and the number of staff interviewed along with their positions.

Location	ANM*	GNM**	PHARMACIST	OTHERS#	TOTAL
**PRIMARY HEALTH CENTRES**					
**Shirsuphal**	1			1	2
**Lasurne**	1	1	1		3
**Kalas**	1				1
**Sanasar**	2			1	3
**Dorlewadi**	1		1	2	4
**Hol**	1	2	2	1	6
**Murti**			2		2
**Parandare**	1		1	1	3
**Kedgaon**		3			3
**Sangavi**	2		1		3
**Moregoan**	1	2	1	1	5
**Katewadi**	1	1	1	1	4
**Loni Bhapker**		2		1	3
**Parwadi**		2	1	1	4
**SUB-CENTRES**					
**Gojubawi**		1			1
**Dalaj**		2			2
**Katphal**	1	1	1		3
**Pimpli**		1	1		2
**TOTAL**	13	18	13	10	54

*ANM- Auxiliary Nursing Midwives,

**General Nursing Midwives

^#^ Others comprised MPW (Multi-purpose worker), Laboratory Technicians, Pharmacists, LHV (Ladies’ Health visitor), HW (Health worker) and OJT (On Job Trained) worker.

The number of years in service for the employees ranged from one to 34 years (average 14 years, median 11 years). Twelve staff members were educated up to university graduate level or higher. None of the participants had received any formal training for the management of injuries sustained from animal bites. All respondents identified dogs as the most common source of animal-bite cases presented to the health centres. Most respondents (53, 98%) said that their centres were equipped to provide PEP treatments to dog-bite victims. Forty-nine (91%) health workers said that their centres were supplied with Anti Rabies Vaccines (ARV), 4 with ARV and RIG, while one respondent said their centre received none. The majority of the participants (51, 94%) reported that ARV was readily available from chemist stores close to their clinic. In contrast only 6 (11%) respondents said that RIG was available in nearby chemist stores.

The bivariate analyses of the responses to the questions on knowledge and the attitudes and practices are presented in Tables 2 and 3, respectively.

**Table 2:** Bivariate analyses of the individual questions pertaining to knowledge about rabies for health workers belonging to different categories (n = 54).

Questions	n = 54 (%)	Years in service			Education			Appointment		
		>11 years (%)	≤ 11 years (%)	p-value	Graduate (%)	Not Graduate (%)	p-value	Nursing (%)	Non-nursing (%)	p-value
Have you heard about rabies?										
Yes	52 (96)	26 (50)	26 (50)	0.5	12 (23)	40 (77)	0.9	30 (57)	22 (43)	0.9
No	2 (4)	0	2 (100)		0	2 (100)		1 (50)	1 (50)	
Do you think rabies can spread to another human from a human patient with rabies?										
Yes	19 (35)	4 (21)	15 (79)	0.004*	7 (37)	12 (63)	0.1	9 (47)	10 (53)	0.3
No	35 (65)	22 (63)	13 (37)		5 (14)	30 (86)		22 (63)	13 (37)	
Do you think rabies can be spread through licks/scratches from an animal?										
Yes	29 (54)	12 (41)	17 (59)	0.3	6 (21)	23 (79)	0.9	21 (72)	8 (28)	0.02*
No	25 (46)	14 (56)	11(44)		6 (24)	19 (76)		10 (40)	15 (60)	
Do you think rabies can be spread through contaminated food or water?										
Yes	13 (24)	6 (46)	7 (54)	0.9	4 (9)	9 (91)	0.4	11 (85)	2 (15)	0.02*
No	41 (76)	20 (49)	21(51)		8 (19)	33 (81)		20 (49)	21 (51)	
Do you think death is inevitable if a person bitten by a rabid animal develops signs of the disease?										
Yes	47 (87)	23 (49)	24 (51)	0.9	11 (48)	36 (52)	0.9	28 (59)	19 (41)	0.4
No	7 (13)	3 (43)	4 (57)		1 (14)	6 (86)		3 (43)	4 (57)	

*Significant p—values.

**Table 3:** Bivariate analyses of the individual questions pertaining to practices of health workers towards management of dog-bite wounds belonging to different categories (n = 54) that help control rabies.

Questions	n = 54 (%)	Years in service		p-value	Education		p-value	Appointment		p-value
		>11 years (%)	≤11 years (%)		Graduate (%)	Not Graduate (%)		Nursing (%)	Non Nursing (%)	
Do you think traditional treatment is useful?										
Yes	2 (4)	1 (50)	1 (50)	0.9	1 (50)	1 (50)	0.4	1 (50)	1 (50)	0.9
No	52 (96)	25 (48)	27 (52)		11 (21)	41 (79)		30 (57)	22 (43)	
Is washing the dog-bite wound with soap water useful?										
Yes	53 (98)	25 (47)	28 (53)	0.5	12 (23)	41 (77)	0.9	31 (58)	22 (42)	0.4
No	1 (2)	1 (100)	0		0	1 (100)		0	1 (100)	
How long do you wash the wound with soap-water?										
>10–15 min	9 (17)	7 (78)	2 (22)	0.1	2 (22)	7 (78)	0.9	6 (67)	3 (33)	0.7
<10–15 min	45 (83)	19 (42)	26 (58)		10 (28)	35 (72)		25 (55)	20 (45)	
Would you suture a dog-bite wound?										
Yes	11 (20)	10 (91)	1 (9)	0.001*	0	11 (100)	0.1	8 (73)	3 (27)	0.3
No	43 (80)	16 (37)	27 (63)		12 (28)	31 (72)		23 (53)	20 (47)	
Do you think it is important to observe a dog that has bitten someone?										
Yes	53 (98)	26 (49)	27 (51)	0.9	12 (23)	41 (77)	0.9	31 (58)	22 (42)	0.4
No	1 (2)	0	1 (100)		0	1 (100)		0		
For how many days should the dog that has bitten someone be observed for?^										
≥10days	42 (78)	22 (52)	20 (48)	0.5	9 (26)	31 (74)	0.7	18 (41)	25 (59)	0.9
<10days	11 (22)	4 (36)	7 (67)		3 (27)	8 (73)		5 (45)	6 (55)	
Are you aware of the schedule of the ARV followed at your clinic?										
Yes	47 (87)	23 (49)	24 (51)		8 (17)	39 (83)		28 (59)	19 (41)	0.4
No	7 (13)	3 (43)	4 (57)	0.9	4 (57)	3 (43)	0.04*	3 (43)	4 (57)	
Should RIG be administered immediately after the dog bite?										
Yes	14 (26)	7 (50)	7 (50)		3 (21)	11 (79)		11 (78)	3 (22)	0.1
No	40 (74)	19 (48)	21 (52)	0.9	9 (23)	31 (77)	0.9	20 (50)	20 (50)	
Can RIG be administered 7 days after the dog bite exposure?										
Yes	41 (76)	17 (41)	24 (59)		10 (24)	31 (76)		21 (51)	20 (49)	0.1
No	13 (24)	9 (69)	4 (31)	0.1	2 (15)	11 (85)	0.7	10 (77)	3 (23)	

*Significant p—values,

^—answers only included for those respondents who believed it was necessary to observe a dog that had bitten someone

The reasons given for the failure to control rabies included a lack of control of free-roaming dogs (FRD) (24, 44%); lack of awareness about the disease by residents (19, 35%); a combination of both lack of awareness and lack of control of FRD (10, 19%); or the non-availability of PEP (1, 2%). Staff with ≤ 11 years of service were less informed about human-to-human transmission of rabies (OR 0.2, 95%CI 0.03–0.6, p = 0.004) than more experienced staff. In contrast, the years of service did not affect responses to other knowledge questions (Table 2). Nursing staff were more aware that rabies could be transmitted by licks and scratches from a rabid animal (OR 3.8, 95%CI 1.0–14.4) and that it could not be transmitted through contaminated food and water (OR 5.6, 95%CI 1.0–58.2, p = 0.02) (Table 2) than the non-nursing staff. Less experienced staff (≤ 11 years of medical service) were more likely to suture a dog-bite wound (OR 16, 95%CI 2.0–755) and graduates were less likely to know the schedule of administration of PEP (OR 0.2, 95%CI 0.02–1.0) (Table 3).

The results of the univariable analyses for the independent variables (years in service, education and appointment of the respondents) with the knowledge of rabies and the practices of the primary health staff to control rabies are summarised in Table 4. The predictors, years in service and appointment, yielded p-values ≤ 0.25 (0.1 and 0.14 respectively) and were offered into the multivariable logistic regression model for assessing the knowledge of the respondents (Table 5). The final model was a good fit of the data (Hosmer-Lemeshow goodness of fit test χ^2^ = 0.71, df = 1, p-value = 0.39).

**Table 4:** Test of association (χ^2^) of the independent variables (experience, education and appointment) with the dependent variables (knowledge about rabies and practices regarding management of dog-bite patients).

**Criteria/variable**	n = 54 (%)	**Knowledgeable respondents (%)**	OR	p-value
**Years in service**				
≤ median#	28 (52)	21 (75)	1	
> median	26 (48)	14 (54)	0.4 (0.1–1.2)	0.1*
**Educational qualifications**				
Graduate	12 (22)	9 (75)	1	
Non-graduate	42 (78)	26 (62)	0.5 (0.1–2.3)	0.5
**Appointment**				
Nursing	31 (57)	23 (74)	1	
Non-nursing	23 (43)	12 (52)	0.4 (0.1–1.2)	0.15*
**Criteria/variable**	n = 54 (%)	**Respondents with positive practices (%)**	OR	p-value
**Years in service**				
≤ median	28 (52)	16 (57)	1	
> median	26 (48)	10 (38)	0.5 (0.2–1.4)	0.2*
**Educational qualifications**				
Non Graduates	42 (78)	23 (55)	1	
Graduates	12 (22)	3 (25)	0.4 (0.07–1.2)	0.1*
**Appointment**				
Nursing	31 (57)	17 (55)	1	
Non-nursing	23 (43)	9 (39)	0.5 (0.2–1.6)	0.2*

*Variables offered to the multivariable models

^#^ Median years of service = 11 years

**Table 5:** Final multivariable model showing the influence of various independent factors over the knowledge about rabies and practices pertaining to rabies that help its control.

**Multivariable model (Knowledge)**				
	Intercept (b)	SE	p- value	OR
**Constant**	-1.8	0.61	_	_
Years in Service*				
≤ median	_	_	_	1
> median	1.2	0.64	0.05	3.4 (1.0–12.1)
***Appointment***				
Nursing	_	_	_	1
Others	1.2	0.64	0.05	3.5 (1.0–12.3)
Likelihood ratio (χ^2^) test = 6.7; p = 0.03; Hosmer-Lemeshow goodness of fit test- χ^2^ = 0.71,df = 1, p-value = 0.39				
**Multivariable model (Practices)**				
	Intercept (b)	SE	p- value	OR
**Constant**	2.05	0.86	_	_
Years in Service*				
≤ median	_	_	_	1
> median	1.2	0.62	0.06	3.2 (1.0–11.0)
***Educational qualifications***				
Non-graduate	**_**	_	_	1
Graduate	1.7	0.8	0.03	5.6 (1.2–27.0)
Likelihood ratio (χ^2^) test = 7.3; p = 0.02;Hosmer-Lemeshow goodness of fit test- χ^2^ = 1.66, df = 1, p-value = 0.19				

* Median years of service = 11 years

The practices towards dog-bite wound management were assessed by developing a multivariable logistic regression model that initially included all the three predictor variables namely, years in service (p = 0.2), appointment (p = 0.2) and educational qualifications (p = 0.1). The final model indicates that more experienced staff and those with higher education adopt better practices (OR 3.2 and 5.6 respectively, Table 5). The final model was found to be a good fit of the data (Hosmer-Lemeshow goodness of fit test χ^2^ = 1.66, df = 1, p-value = 0.19).

## 4 Discussion

Paramedical staff are usually the first point of interaction for a dog-bite victim in rural India. However few studies have been conducted in India focusing on the KAP of these staff at PHC and sub-centres. Although one would hope that the frontline health staff in rural areas are specifically trained on rabies and animal bite management, this study found that none of the health staff surveyed had received formal training on the management of dog-bite related injuries, hence, it is not surprising that awareness about some aspects of rabies-control and treatment of dog-bites was low.

Most respondents (96%) were aware of the disease, but this did not equate to knowledge on the best method of prevention or the correct protocol for administering PEP/RIG. In contrast to a recent study conducted in the rural areas of Himachal Pradesh in northern India, reporting irregular supply or total lack of ARV [20], we found that ARV was available at all centres surveyed and RIG at three of the 18 centres. It was apparent that not all staff surveyed were aware of the availability of preventive measures against rabies in their clinic as one respondent reported that they had neither ARV nor RIG in their centre, even though the other respondent from the same centre reported receiving regular supplies. The improved availability of PEP in areas around Baramati may be attributed to effective implementation of the government’s initiatives after the 12^th^ Five year plan and better practical application of these measures in the area of study compared to some other parts of India [21]. Five year plans are regular economic policy roll-outs in India and rabies was included as a disease of economic consequence for the first time in 2012. Additionally, easier accessibility to big commercial cities viz. Pune compared to some other parts of India that are located further from commercial centres, could also be a factor for availability of PEP in the area of this study. The high percentage (94%) of respondents confirming the availability of ARV in nearby private medical stores supports the increased awareness of PEP in this study area. However, it also indicates that there are a high number of dog-bite injuries in the area [22].

In this study, 24% of staff believed that rabies could be transmitted through contaminated food or water or between humans (an extremely rare possibility [1]). Although only approximately half (54%) of the participants were not aware that rabies could be transmitted through licks and scratches of a rabid animal, this was an improvement from a similar study conducted in north India, where 80% of nursing staff and 73% of non-nursing staff did not know other modes of transmission of rabies other than animal bites [11]. Another study in Vietnam of public health workers similarly reported poor awareness of the potential risk from licks/scratches of rabid animals [23]. These findings highlight the need for a prescribed training module on the management of animal bite cases and rabies for frontline health staff.

Although most health staff were aware of the need to wash a bite wound with soap and water, a practice that can reduce human rabies cases by at least 65% [13], a majority (83%) would wash the wound for less than ten minutes, while the recommended duration is more than 15 minutes [4, 24]. Similarly, although almost all the health staff were aware that a dog that had bitten someone should be observed, only half of them correctly reported that the observation should be for 10 days [25]. These features are important in the transmission of rabies and if information about them are correctly disseminated amongst the PHC staff, the incidence of rabies could be substantially reduced.

While all the staff interviewed said that they were aware of the treatment to be given to a dog-bite victim, only 87% could actually correctly state the schedule of vaccination to be followed. It has also been reported that even physicians practicing in rural India are not adequately informed about the importance of administering vaccinations and immunoglobulins in cases of any dog-bite injury, regardless of severity [2]. However, more than half of the staff that were unaware of the ARV schedule (57%) in the current study belonged to the non-nursing appointments. Not unexpectedly, given the low availability of RIG in the area, few health staff surveyed were aware of RIG and its administration.

Generally, the more experienced workers and non-nursing staff were more knowledgeable about the practices (OR 3.4 and 3.5, respectively) required to control rabies, as indicated by the multivariable model (Table 5), although contrasting results were obtained in the univariable analyses (Table 4). Similarly, the multivariable model for practices indicates that more experienced workers and better-educated staff adopt better practices towards dog-bite injury management (OR 3.2 and 5.6) in contrast to the findings of univariable analyses. This dichotomy may be peculiar to this study because half of the respondents who had less experience were comprised of non-nursing staff (who were better educated) thus having a better knowledge score. Although not significant at the 95% confidence level, the odds of an experienced worker being a nursing staff was higher (OR 1.8) and the odds of an experienced worker being better educated was also significant at the 90% confidence level (OR 3.5, p = 0.07). We surmise that such opposing results may be atypical of this data set and can be reduced in studies with larger sample sizes. However, knowledge gaps were identified even in experienced staff as they were more likely to favour the suturing of an animal bite wound (OR 16.1, p = 0.001), contrary to the recommended practice of avoiding suturing animal-bite wounds or to suture only after instilling RIG [26]. Such inconsistencies could be overcome by mandatory training of health care staff at the commencement of their service, in conjunction with periodic training updates during their career.

In contrast, the knowledge about rabies of the non-nursing staff was found to be comparatively better (OR 3.5) than the nursing staff. This can be explained by the fact that most of the non-nursing staff were pharmacists and laboratory technicians and the majority of them (68%) were graduates with presumably more theoretical knowledge about rabies. The non-graduate nursing staff (Auxiliary Nursing Midwives and General Nursing Midwives) who are trained to perform nursing procedures, had a lower level of knowledge on animal-bite injuries and rabies. The poorer practices adopted by non-graduates (predominantly nursing staff) could be improved by initial and regular training.

In summary, we found that staff in the early period of their service and those who are not graduates lacked comprehensive knowledge on rabies, and reflect poor perception regarding practices that can reduce the incidence of dog-bite related rabies (Table 5).

As some non-nursing staff were found to treat dog-bite injuries in the absence of nursing staff, it is similarly important that these staff should also be included in training on rabies and dog-bite wound management.

The ubiquitous presence of unrestricted FRD was cited by 44% of the respondents for the presence of rabies in the area of study, followed by a lack of awareness about rabies in the general population (35%). This reiterates the WHO policy for control of rabies in developing countries where emphasis has been placed on managing the FRD population, along with mass vaccination of FRD [27]. The role of education in the control of zoonotic diseases has been emphasised by Robertson, Irwin [28] and Slater [29], and rabies is no exception. There is a need to develop educational campaigns for the general public as well, and these campaigns should include information on the need to seek immediate treatment for dog-bites and to not rely on traditional methods of treating dog-bite wounds. This is also an indicator that knowledge about rabies among health staff in rural PHC and sub-centres is not complete, as noted by the lack of a stipulated observation period of dogs. Education campaigns should also emphasise that there is no correlation between the period of observation of a dog involved in a biting incident, and administration of PEP, and PEP should be sought immediately after any animal bite.

It is likely that more remote centres would have different outcomes than that found in this study [11]. It would be useful to expand the study to all staff in the centres and to sample further rural centres to ensure findings were consistent across a larger area of rural India when developing control measures and educational packages.

Our study highlights the need for programs to ensure that staff dealing with dog-bite victims have correct knowledge about rabies and know how to correctly treat such injuries to reduce the incidence of rabies in rural India.

## Supporting information

S1 File Excel sheet. (XLSX)

S2 File Appendix—Questionnaire. (PDF)
