# Supplementary material for: Knowledge, attitudes and practices towards dog-bite related rabies in para-medical staff at rural primary health centres in Baramati, western India
Source: PLoS One. 2018 Nov 16;13(11):e0207025. doi: 10.1371/journal.pone.0207025 (PMC6239288; doi:10.1371/journal.pone.0207025)
Supplement: S2 File — (PDF) [file pone.0207025.s002.pdf]

C. ____

Panchayat Samiti Baramati
Taluka health office
Outward no.health/ /2016
Baramati- date- 15/11/2016

TO

Ashoka Trust for Research in
Ecology and the Environment.

SUBJECT- PERMISSION TO CONDUCT KAP SURVEY FOR PRIMARY MEDICAL STAFF ON DOG BITE CASE MANAGEMENT.

REFERENCES- Refer to your request letter dated 11/11/2016 from ATREE.

I hereby grant permission to ATREE staff Lt. Col. Dr. Harish Tiwari and his team to conduct questionnaire survey of the health staff at primary /block /town level health centres. You are also hereby allowed to collect data pertaining to dog bites /rabies cases from respective centres for last two years.

Taluka health officer
Panchayat Samiti Baramati

Division of Research & Development
Research Ethics and Integrity

Chancellery Building
South Street
MURDOCH WA 6150
Telephone: (08) 9360 6677
Facsimile: (08) 9360 6686
human.ethics@murdoch.edu.au

www.murdoch.edu.au

Thursday, 05 May 2016

Prof Ian Robertson
School of Veterinary and Life Sciences
Murdoch University

Dear Ian,

Project No. 2016/020
Project Title Studies on the population dynamics of free roaming stray dogs and owned dogs in Mumbai and Guwahati

Thank you for addressing the conditions placed on the above application to the Murdoch University Human Research Ethics Committee. On behalf of the Committee, I am pleased to advise the application now has:

OUTRIGHT APPROVAL

Approval is granted on the understanding that research will be conducted according the standards of the ***National Statement on Ethical Conduct in Human Research* (2007), the *Australian Code for the Responsible Conduct of Research* (2007)** and **Murdoch University policies** at all times. You must also abide by the **Human Research Ethics Committee's standard conditions of approval (see attached).** All reporting forms are available on the Research Ethics and Integrity web-site.

I wish you every success for your research.

Please quote your ethics project number in all correspondence.

Kind Regards,

Dr. Erich von Dietze
Manager
Research Ethics and Integrity

cc: Dr Mark O'Dea and Harish Tiwari

Human Research Ethics Committee: Standard Conditions of Approval

a) The project must be conducted in accordance with the approved application, including any conditions and amendments that have been approved. You must comply with all of the conditions imposed by the HREC, and any subsequent conditions that the HREC may require.
b) You must report immediately anything, which might affect ethical acceptance of your project, including:
   - *Adverse effects on participants*
   - *Significant unforeseen events*
   - *Other matters that might affect continued ethical acceptability of the project.*
c) Proposed changes or amendments to the research must be applied for, using an Amendment Application form, and approved by the HREC before these may be implemented.
d) An Annual Report for the project must be provided by the due date specified each year (usually the anniversary of approval).
e) A Closure Report must be provided at the conclusion of the project (once all contact with participants has been completed).
f) If, for any reason, the project does not proceed or is discontinued, you must advise the committee in writing, using a Closure Report form.
g) If an extension is required beyond the end date of the approved project, an Extension Application should be made allowing sufficient time for its consideration by the committee. Extensions of approval cannot be granted retrospectively.
h) You must advise the HREC immediately, in writing, if any complaint is made about the conduct of the project.
i) Other Murdoch approvals (e.g. fieldwork approval) or approval form other institutions may also be necessary before the research can commence.
j) Any equipment used must meet current safety standards. Purpose built or modified equipment must be tested and certified by independent experts for compliance with safety standards.
k) Graduate research degree candidates must normally have their Program of Study approved prior to commencing the research. Exceptions to this must be approved by the HREC.
l) You must notify Research Ethics & Integrity of any changes in contact details including address, phone number and email address.
m) Researchers should be aware that the HREC may conduct random audits and / or require additional reports concerning the research project.

Failure to comply with the *National Statement on Ethical Conduct in Human Research* (2007) and with the conditions of approval may result in the suspension or withdrawal of approval for the project.

The HREC seeks to support researchers in achieving strong results and positive outcomes.

The HREC promotes a research culture in which ethics is considered and discussed at all stages of the research.

If you have any issues you wish to raise, please contact the Research Ethics Office in the first instance.

प्राथमिक आरोग्य अभ्यासाचे सर्वेक्षण

प्रोजेक्ट टाईटल: प्राथमिक आरोग्य केंद्रे / क्लिनिक / रुग्णालये यांच्यामध्ये कुत्र्याशी संबंधित रेबीजकडे काम करणा-या आरोग्य कर्मचा-यांचे ज्ञान, वृत्ती आणि प्रथा (केएपी) निश्चित करण्यासाठी एक सर्वेक्षण.

मर्डोक विद्यापीठातील पीएचडी विद्यार्थिनी हरिश कुमार तिवारी यांनी प्राथमिक आरोग्य कर्मचा-यांना मोफत रोमी कुत्री, कुत्री-आधारित रेबीज आणि प्रथमोपचार पद्धतींचा अभ्यास, ज्ञान, वर्तणूक आणि प्रथा शिकवल्या. हा अभ्यास कॉलेज ऑफ व्हेटर्नरी मेडिसिन, मर्डोक युनिव्हर्सिटी आणि अशोका ट्रस्ट फॉर रिसर्च ऑन पारिस्थितिकी आणि पर्यावरण, बेंगलोर, कर्नाटक, भारत यांच्यातील सहयोगी अभ्यास आहे.

या अभ्यासाचा मुख्य हेतू म्हणजे प्राथमिक आरोग्य अधिकाऱ्यांमधील जागरूकता वाढवणे हे सहसा कुत्रा-पीडित व्यक्तीचे संपर्क करणारे पहिले बिंदू आहेत. हे भारतातील कुत्र्यांशी संबंधित रेबीजसाठी चांगले नियंत्रण उपाय शोधण्यात मदत करेल आणि प्राथमिक आरोग्य कर्मचार्यांची भूमिका या रोगाविरोधात लढण्यात निश्चित करेल.

या सर्वेक्षणात आपला सहभाग खूप कौतुक होईल. सर्वेक्षण पूर्ण करण्यासाठी सुमारे 30 मिनिटे लागतील. सर्वेक्षणाचा एक भाग म्हणून काही प्रश्नांना आपल्या सराव व व्यावसायिक तपशीलांशी संबंधित माहिती आणि रेबीज विषयी आपले ज्ञान, वृत्ती आणि प्रथांशी संबंधित प्रश्न असतील.

आपल्याकडून संकलित केलेली सर्व माहिती अतिशय गोपनीय ठेवली जाईल. आपण ओळखू शकणारी कोणतीही माहिती कोणत्याही अहवालात किंवा प्रकाशनात वापरली जाणार नाही. आपला सहभाग संपूर्णपणे स्वयंसेवी आहे. आपण कोणत्याही प्रभाव न करता प्रश्नावलीमधून कधीही मागे घेऊ शकता.

संमती:

या अभ्यासाबद्दल आपल्याकडे काही प्रश्न आहेत का? होय नाही

आपण अभ्यासाचा आणि आपल्या सहभागाचा उद्देश समजून घेता का? होय नाही

आपण अभ्यासात सहभागी होऊ इच्छिता? होय नाही

कृपया साइन इन करा म्हणजे आम्ही या अभ्यासात भाग घेण्यासाठी आपली संमती रेकॉर्ड करू या सर्वेक्षणाशी संबंधित काही शंका किंवा चिंता असल्यास आपण मर्डोक विद्यापीठातील मानवी नैतिक कार्यालयाशी संपर्क साधू शकता किंवा मानवीय ईमेल येथे संपर्क साधू शकता.
Ethics@murdoch.edu.au.

या प्रकल्पाच्या सहाय्याने आपल्या मदतीबद्दल धन्यवाद.
प्रामाणिकपणे

Harish Kumar Tiwari PhD Student
School of Veterinary
and Life Sciences
Murdoch University
South Street,
Murdoch 6150
Western
Australia
Phone : 0426842710
Email: h.tiwari@murdoch.edu.au

रेबीज: प्राथमिक आरोग्य केंद्रामध्ये ज्ञान, स्वभाव आणि व्यवहार

1. Demographic details/डेमोग्राफिक तपशील

1.1	What is your highest medical qualification?<br>तुमची सर्वोच्च वैद्यकीय योग्यता कोणती आहे?	
1.2	How many years have you been working in the health profession?<br>आपण आरोग्यासाठी किती वर्षे काम करीत आहात व्यवसाय?	
1.3	Have you undergone any special training pertaining to the management of animal bite cases?<br>आपण कोणत्याही पशु चाव्याव्दारे खटल्यांचे व्यवस्थापन संबंधित विशेष प्रशिक्षण संबंधित आहेत काय ?	Yes( ) No ( )

1. 2. **Knowledge, attitudes and practices about rabies/** रेबीज विषयी ज्ञान, वृत्ती आणि पद्धती

2.1	Which animal is responsible for the most cases of bite injuries presented to your practice/clinic?<br>कोणता प्राणी जखमेच्या बहुतांश प्रकरणांसाठी आपल्या क्लिनिकमध्ये आलेल्या दुखापत?	
2.2	Have you heard of rabies? If yes, please proceed to next question. If no, please proceed to 2.9<br>आपण रेबीजबद्दल ऐकले आहे का?	Yes ( ) No ( )
2.3	Do you think rabies can be spread to another human from a human patient with rabies?<br>तुम्हाला असे वाटते की रेबीज इतर मनुष्यांपर्यंत पसरला जाऊ शकतो काय?	Yes ( ) No ( )<br>Not Sure ( )
2.4	Do you think rabies can be spread through the bite of an animal?<br>तुम्हाला वाटते रेबीज एखाद्याच्या प्राणीचाव्याव्दारे पसरू शकतात काय?	Yes ( ) No ( )<br>Not Sure ( )
2.5	Do you think rabies can be spread through licks or scratches from an animal?<br>आपण रेबीज एक प्राणी पासून licks किंवा scratches माध्यमातून पसरली जाऊ शकते वाटते?	Yes ( ) No ( )<br>Not Sure ( )
2.6	Do you think rabies can be spread through contaminated food or water?<br>तुम्हाला असे वाटते की रेबीज दूषित अन्न किंवा पाणी होण्याव्दारे पसरतात?	Yes ( ) No ( )<br>Not Sure ( )
2.7	Do you think death is inevitable if a person bitten by a rabid animal develops signs of rabies?<br>एखादी व्यक्ती प्राणी रेबीजची चिन्हे विकतात असेल तर मृत्यूचा अपरिहार्य?	Yes ( ) No ( )<br>Not sure ( )

2.8	Do you think a person bitten by a rabid animal can be saved from developing rabies?<br>आपण विचार करतो की एखाद्या पाशवी वृत्तीने बाधा मारलेली व्यक्ती रेबीज विकसित करण्यापासून वाचू शकते?	Yes ( ) No ( )<br>Not Sure ( )
2.9	What is the most common treatment given to a patient bitten by a dog at your clinic?<br>रेबीज विकसित करण्यापासून आपल्या क्लिनिकमध्ये एखाद्या कुत्र्याचा वापर करून रुग्णाला दिलेला सर्वात सामान्य उपचार काय आहे?	Apply local treatment like chilli powder/ turmeric<br>( )<br>Apply antiseptic cream/ powder<br>( )<br>Wash with soap/ detergent<br>( )<br>Apply antibiotics<br>( )<br>Other, please specify,<br>( )
2.10	Do you think the treatment of an animal bite wound with chilli/ turmeric powder is useful? आपल्याला असे वाटते की एखाद्या प्राण्याशी संबंधित काचेचे जखमेच्या उपचाराने मिरची / हळद पावडर उपयुक्त आहे का?	Yes ( ) No ( )<br>Not sure ( )
2.11	Do you think washing an animal bite wound with soap/ detergent and water is useful? If yes, proceed to 2.12, else to 2.13<br>तुम्हाला वाटते साबण / डिटर्जंट आणि पाण्याचा वापर करून प्राण्यांच्या चाव्यावर जखम करणे उपयुक्त आहे?	Yes ( ) No ( )<br>Not sure ( )
2.12	When the wound is washed with soap/detergent and water, how long do you think it should take to complete the procedure?<br>जेव्हा साबण / डिटर्जंट आणि पाण्याने जखमा केला जातो तेव्हा ते प्रक्रिया पूर्ण होण्यासाठी किती वेळ लागेल?	< 2 minutes ( )<br>2 – 5 Minutes ( )<br>6 – 10 Minutes ( )<br>11 – 15 Minutes ( )<br>>15 Minutes ( )
2.13	In your clinic would you suture a wound caused by a dog bite? आपल्या क्लिनिकमध्ये आपण एखाद्या कुत्रेमुळं जखमा काढला असता कापणे?	Yes ( ) No ( )<br>Sometimes ( )
2.14	Do you think it is important to observe a dog that has bitten someone? If no, proceed to 2.16<br>कुणीतरी कुत्र्याची चाट पाहिली पाहिजे असं तुम्हाला वाटतं का?	Yes ( ) No ( )<br>Not sure ( )
2.15	How many days do you think that the dog that has bitten a person or animal should be observed for? तुला असे वाटते की कुत्राला कात टाकली आहे व्यक्ती किंवा प्राणी साठी किती वेळा साजरा पाहिजे?	
2.16	Do you know of treatments that can be given to a person bitten by a rabid animal to prevent rabies? If yes, proceed to next question, else thanks for your time to complete this questionnaire. रेबीज टाळण्यासाठी एखाद्या प्राणघातक प्राण्याद्वारे वापरलेल्या एखाद्या व्यक्तिस दिलेल्या उपचारांबद्दल तुम्हाला माहिती आहे का?	Yes ( ) No( )<br>Not Sure ( )

2.17	Are such treatments that can prevent rabies after an animal bite administered at your clinic? If yes, proceed to next question , else to 2.19<br>अशा क्लिनिकमध्ये जनावरांना चावणे झाल्यानंतर अशा प्रकारच्या उपचारांमुळे रेबीजला प्रतिबंध करता येतो का?	Yes ( ) No( )<br>Not Sure ( )
2.18	What PEP (Post exposure prophylaxis) is available at your clinic?<br>आपल्या क्लिनिकमध्ये कोणत्या पीईपी (पोस्ट एक्सपोजर प्रॉफिलॅक्सिस) उपलब्ध आहे?	Anti-rabies vaccine(ARV) _____<br>RIG (rabies immunoglobulins) _______<br>Both ( ) None ( ) NA ( )
2.19	Is anti-rabies vaccine (ARV) readily available from the medical stores around this area when required?<br>अँटी-रेबीज लस (एआरव्ही) आवश्यकतेनुसार या क्षेत्राबाहेरील वैद्यकीय स्टोअर्स हे तात्काळ उपलब्ध आहे ?	Yes ( ) No ( )<br>Not sure ( )
2.20	Is rabies immunoglobulin (RIG) readily available from the medical stores when required?<br>रेबीज इम्युनोग्लोब्युलिन (आर.आय.जी.) हे आवश्यक तेव्हा वैद्यकीय स्टोअर्स सहजगत्या उपलब्ध आहे?	Yes ( ) No ( )<br>Not sure ( )
2.21	Are you aware of the schedule of ARV to follow? If no, please proceed to 2.23<br>आपण अनुसरून एआरव्हीचे schdule माहित आहात?	Yes ( ) No ( )<br>Not sure ( )
2.22	What is the schedule of ARV you follow in your clinic?<br>आपण एआरव्हीचे अनुसरून schdule माहित आहात?	
2.23	Do you think RIG should be administered immediately after a person is bitten by an animal?<br>आपल्याला असे वाटते की एखाद्या व्यक्तीला एखाद्या प्राण्याने लगेच ताबडतोब RIG प्रशासित केले पाहिजे जातो का?	Yes ( ) No ( )<br>Not sure ( )
2.24	Do you think RIG can be administered up to 7 days after exposure? तुम्हाला असे वाटते का, एखाद्या व्यक्तीला एखाद्या प्राण्याने 7 दिवसांपुर्वी RIG चा वापर केला जाऊ शकतो का?	Yes ( ) No ( )<br>Not sure ( )
2.25	In your opinion which is the most important factor that results in failure to control rabies in humans?<br>आपल्या मते ही मानवातील रेबीजवर नियंत्रण ठेवण्यात अपयश येणारे सर्वात महत्त्वाचे घटक आहे?	Non availability of ARV/ PEP/RIG ( )<br>Lack of awareness among people ( )<br>Lack of control over stray dog population( )<br>Others please specify<br>( )

ही प्रश्नावली पूर्ण करण्याच्या आपल्या वेळेसाठी धन्यवाद. ही माहिती रेबीज समजण्यास मदत करेल आणि भारतात त्याच्या नियंत्रणास मदत करेल.

Consent form for the Survey of Primary Health Staff

Project Title: A survey to determine the knowledge, attitudes and practices (KAP) of health workers employed with Primary Health Centres/ Clinics/ hospitals towards canine related rabies.

Harish Kumar Tiwari, a PhD student at Murdoch University is studying the knowledge, attitudes and practices of primary health workers towards free roaming dogs, dog associated rabies and the first aid measures adopted. This study is a collaborative study between the College of Veterinary Medicine, Murdoch University and Ashoka Trust for Research on Ecology and Environment, Bangalore, Karnataka, India.

The main objective of the study is to ascertain the level of awareness among primary health officials who are generally the first point of contact for a dog-bite victim. It will help identify better control measures for dog related rabies in India and determine the role of the primary health workers in the fight against this disease.

Your participation in this survey will be greatly appreciated. The survey will take about 30 minutes to complete. As part of the survey some questions will relate to information regarding your place of practice and professional details followed by questions relating to your knowledge, attitudes and practices towards rabies.

All information collected from you will be kept strictly confidential. No information that may identify you will be used in any report or publication. Your participation is entirely voluntary. You may withdraw at any time from the questionnaire without any impact on you.

Consent:

Do you have any questions about this study?	Yes	No
Do you understand the purpose of the study and your involvement?	Yes	No
Would you like to participate in the study?	Yes	No

Please sign so that we record your consent to participate in this study ____________________

If you have any queries or concerns regarding this survey you can contact Human ethics office at Murdoch University or email at Human.Ethics@murdoch.edu.au.

Thank you for your assistance with this project.

Sincerely

Harish Kumar Tiwari
PhD Student
School of Veterinary and Life Sciences
Murdoch University
South Street, Murdoch 6150
Western Australia
Phone : 0426842710
Email: h.tiwari@murdoch.edu.au

Rabies: Knowledge, attitudes and practices at primary health centres

1. Demographic details

1.1	What is your highest medical qualification?	
1.2	How many years have you been working in the health profession?	
1.3	Have you undergone any special training pertaining to the management of animal bite cases?	Yes( ) No ( )

2. Knowledge, attitudes and practices about rabies

2.1	Which animal is responsible for the most cases of bite injuries presented to your practice/clinic?	
2.2	Have you heard of rabies? If yes, please proceed to next question. If no, please proceed to 2.9	Yes ( ) No ( )
2.3	Do you think rabies can be spread to another human from a human patient with rabies?	Yes ( ) No ( )<br>Not Sure ( )
2.4	Do you think rabies can be spread through the bite of an animal?	Yes ( ) No ( )<br>Not Sure ( )
2.5	Do you think rabies can be spread through licks or scratches from an animal?	Yes ( ) No ( )<br>Not Sure ( )
2.6	Do you think rabies can be spread through contaminated food or water?	Yes ( ) No ( )<br>Not Sure ( )
2.7	Do you think death is inevitable if a person bitten by a rabid animal develops signs of rabies?	Yes ( ) No ( )<br>Not sure ( )
2.8	Do you think a person bitten by a rabid animal can be saved from developing rabies?	Yes ( ) No ( )<br>Not Sure ( )
2.9	What is the most common treatment given to a patient bitten by a dog at your clinic?	Apply local treatment like chilli powder/ turmeric<br>( )<br>Apply antiseptic cream/ powder<br>( )<br>Wash with soap/ detergent<br>( )<br>Apply antibiotics<br>( )<br>Other, please specify,<br>( )
2.10	Do you think the treatment of an animal bite wound with chilli/ turmeric powder is useful?	Yes ( ) No ( )<br>Not sure ( )
2.11	Do you think washing an animal bite wound with soap/ detergent and water is useful? If yes, proceed to 2.12, else to 2.13	Yes ( ) No ( )<br>Not sure ( )
2.12	When the wound is washed with soap/detergent and water, how long do you think it should take to complete the procedure?	< 2 minutes ( )<br>2 – 5 Minutes ( )<br>6 – 10 Minutes ( )<br>11 – 15 Minutes ( )<br>>15 Minutes ( )
2.13	In your clinic would you suture a wound caused by a dog bite?	Yes ( ) No ( )<br>Sometimes ( )
2.14	Do you think it is important to observe a dog that has bitten someone? If no, proceed to 2.16	Yes ( ) No ( )<br>Not sure ( )

2.15	How many days do you think that the dog that has bitten a person or animal should be observed for?	
2.16	Do you know of treatments that can be given to a person bitten by a rabid animal to prevent rabies? If yes, proceed to next question, else thanks for your time to complete this questionnaire.	Yes ( ) No( )<br>Not Sure ( )
2.17	Are such treatments that can prevent rabies after an animal bite administered at your clinic? If yes, proceed to next question , else to 2.19	Yes ( ) No( )<br>Not Sure ( )
2.18	What PEP (Post exposure prophylaxis) is available at your clinic?	Anti-rabies vaccine(ARV) _____<br>RIG (rabies immunoglobulins) ______<br>Both ( ) None ( ) NA ( )
2.19	Is anti-rabies vaccine (ARV) readily available from the medical stores around this area when required?	Yes ( ) No ( )<br>Not sure ( )
2.20	Is rabies immunoglobulin (RIG) readily available from the medical stores when required?	Yes ( ) No ( )<br>Not sure ( )
2.21	Are you aware of the schedule of ARV to follow? If no, please proceed to 2.23	Yes ( ) No ( )<br>Not sure ( )
2.22	What is the schedule of ARV you follow in your clinic?	
2.23	Do you think RIG should be administered immediately after a person is bitten by an animal?	Yes ( ) No ( )<br>Not sure ( )
2.24	Do you think RIG can be administered up to 7 days after exposure?	Yes ( ) No ( )<br>Not sure ( )
2.25	In your opinion which is the most important factor that results in failure to control rabies in humans?	Non availability of ARV/ PEP/RIG ( )<br>Lack of awareness among people ( )<br>Lack of control over stray dog population( )<br>Others please specify<br>( )

Thank you for your time to complete this questionnaire. This information will help understand rabies and assist in its control in India.
